# Family-wise resistance to infectious salmon anemia suggests that early systemic recognition protects against disease but not viral transcription

**DOI:** 10.3389/fimmu.2026.1805961

**Published:** 2026-05-20

**Authors:** Amber E. Johnston, Deborah A. Bouchard, Demitri Lifgren, Sarah M. Turner, Daniel M. DeLap, Mark P. Polinski

**Affiliations:** 1 Aquatic Animal Health Laboratory, Aquaculture Research Institute, Cooperative Extension, University of Maine, Orono, ME, United States; 2 National Cold Water Marine Aquaculture Center, United States Department of Agriculture, Agricultural Research Service, Orono, ME, United States

**Keywords:** Atlantic salmon, disease resistance, gene expression pathways, infectious salmon anemia (ISA), ISAV, phenotypic resistance

## Abstract

**Introduction:**

Infectious salmon anemia virus (ISAV), the causative agent of infectious salmon anemia (ISA), is one of the most regulated pathogens affecting Atlantic salmon aquaculture globally. Recent investigations suggest a linkage between the host genome and ISA resistance, identifying a potential for promoting resistance through selective breeding. Herein, we sought to explore the dynamics associated with phenotypic ISA and/or ISAV resistance to better mitigate ISA disease.

**Methods:**

Forty families of North American Atlantic salmon were challenged with ISAV to determine family-wise survival, with fish from each family distributed across 16 tanks (one fish per family per tank) and exposed via injection (*n* = 4 per family) or cohabitation (*n* = 12 per family). Four additional tanks (one fish/family/tank) were sampled at either early-onset (11 days post challenge), peak mortality (21 and 35 days post challenge), or resolution (49 days post challenge) phases of disease. Viral load was determined by RT-qPCR and host gene expression was determined by digital PCR in kidney, spleen, heart, and gill. Select spleen samples were also used for RNA sequencing (RNA-seq) transcriptomics.

**Results:**

The most resistant (*N* = 12) and susceptible (*N* = 13) families had a mean cumulative mortality of 34.3% and 78.5%, respectively. Selective breeding for growth and sea lice resistance (30 families) did not notably impact ISA resistance relative to randomly mated control families (10 families). However, ISA and ISAV resistance were not synonymous—phenotypic ISA-resistant and -susceptible fish were similarly burdened with virus in all organs screened. Gene expression and RNA-seq data identified early viral recognition, particularly in the kidney, as a marker for ISA resistance.

**Conclusions:**

Selective breeding for enhanced growth and sea lice resistance did not appear to incidentally impact family-wise resistance to ISA. Phenotypic ISA resistance was at least partially linked to differential gene expression patterns in the kidney, highlighting early upregulation of antiviral, inflammatory, and endothelial growth factors of resistant fish. However, disease (ISA) resistance was not associated with reduced ISAV transcripts, which showed prolonged high loads in the spleen of all families during infection. These data support early recognition of ISAV in kidney as important in disease mitigation, even when ISAV replication is systemic.

## 1 Introduction

Infectious salmon anemia (ISA), caused by infectious salmon anemia virus (ISAV; Family *Orthomyxoviridae*), is one of the most impactful diseases affecting the Atlantic salmon (*Salmo salar*) aquaculture industry with a capacity to cause severe clinical morbidity and mortality (1–3). First detected in Norway in 1984 (4), ISAV has subsequently been found in many leading salmon-producing countries including Chile (5, 6), Scotland (7), Canada (8–10), and the United States (11). Regardless of region, ISAV has led to often devastating losses, with some severe outbreaks surpassing 90% mortality (12). As a result, ISAV is a reportable pathogen to the World Organization of Animal Health and detections can require mandatory depopulation or strict quarantine of affected lots in many jurisdictions (3, 13–15).

In addition to causing significant disease, ISAV is also genetically diverse. Virus isolates are divided into two distinct genotypes known as the European genotype (Genotype 1) and the North American genotype (Genotype 2) (16, 17)—across which there are two known phenotypic variants. Phenotypic variants are based on a highly polymorphic region (HPR) of the hemagglutinin esterase (HE) gene: the virulent HPR-deleted (ISAV HPRΔ) variant and an avirulent “full length” HPR variant (ISAV HPR0) (18). Previous literature has suggested the emergence of the pathogenic HPRΔ from avirulent HPR0 (19, 20), though the exact relationship between the two remains ambiguous and is complicated by the occurrence of both phenotypes in both North American and European genotypes.

Congruent with other orthomyxoviruses, ISAV exhibits high genetic diversity across isolates and likely utilizes epithelial cells as a portal of entry (21). However, once systemic, the virulent ISAV HPRΔ variant has an atypical affinity for endothelial cells rather than respiratory epithelial cells often targeted by other orthomyxoviruses (e.g., influenza viruses in mammals, 21). The tropism of virulent ISAV HPRΔ for endothelial cells and the ability to bind to salmon erythrocytes (both via the 4-*O*-acetylated sialic acid receptor; 22, 23) contribute heavily to the virus’ ability to spread systemically and thus create the potential for dynamic organ distribution and significant variability in host response. Clinical signs of ISAV include significant degeneration and necrosis in multiple organs including the spleen, kidneys, liver, and intestine (2, 5, 24–26). Other hallmarks of ISA include petechial hemorrhaging (both internal and external), gill pallor, exophthalmia, ascites, and clinical anemia (16). ISAV HPRΔ genomic material has been detected in most organs during infection, including the heart, spleen, gill, kidney, and liver where viral loads become highest 8–14 days post infection (16, 27, 28). Taken together, these observations suggest that the early time points following ISAV exposure are critical in shaping host resistance to systemic ISAV replication and disease.

Recent advances in sequencing technologies have encouraged genetic selection for phenotypic disease resistance, and there is global interest in establishing disease-resistant finfish-lines against many aquatic pathogens (29, 30). Current literature suggests high potential phenotypic variability in the Atlantic salmon response to ISAV, opening the door for potential ISA phenotypic resistance research and developing ISAV- or ISA-resistant lines (31, 32). Furthermore, estimated heritability values for survival following ISAV exposure range from 0.13 to 0.33 as determined by two multi-family disease challenges (31, 33). Multiple quantitative trait loci (QTLs) potentially indicative of disease resistance have been identified (34, 35); however, these markers may vary between different stocks of Atlantic salmon—particularly North American and European stocks that have distinct genomic differences (36, 37).

Recent literature suggests a linkage between the host genome and ISA susceptibility in Atlantic salmon. Phenotypic disease resistance has also been explored in resistant and susceptible phenotypes through transcript analysis, with results suggesting linkages between the host transcriptome and disease susceptibility and mortality (32, 33, 38–40) pathways found to be differentially regulated between phenotypically resistant and susceptible fish mostly included those associated with immune regulation such as the interferon system and NLR (nod-like receptor) pathways (39). Ubiquitination, intracellular transport, and inflammation pathways have also been shown to be differentially impacted, although expression patterns vary greatly by organ type (head kidney, spleen, and gill) and time point sampled (7 and 14 days post ISAV exposure) (39). Of note, little has been explored via transcriptomics or gene expression analyses at time points greater than 14 days post challenge (dpc) or in regard to heart tissue, an organ of interest for endothelial cell-targeting viruses such as ISAV (21).

The relationship between ISAV resistance and clinical ISA resistance remains unknown. Herein, we define ISA resistance as an outcome-based phenotype inferred from survival following ISAV challenge and ISAV resistance as resistance to ISAV infection/replication inferred through ISAV genome copies. Some literature has linked ISAV load and/or time to death of the host to clinical ISA severity or disease resistance (38, 41), though it remains unknown if ISA and ISAV resistance occur together. An additional unknown is if/how ISA-resistance-based selective breeding may impact existing breeding programs selecting for other commercially relevant traits (i.e., growth), or *vice versa*.

To this end, a multi-family ISAV cohabitation exposure experiment was conducted to (1) confirm family-wise phenotypic ISA resistance in North American Atlantic Salmon, (2) identify if selective breeding efforts focused on growth and sea lice resistance have incidentally impacted ISA resistance, (3) determine viral load dynamics in multiple tissues to evaluate ISA and ISAV relationships, and (4) compare transcriptional patterns of ISA-resistant and -susceptible phenotypes to identify potential mechanisms and/or markers of resistance.

## 2 Materials and methods

### 2.1 Fish and experimental environment

Forty single-year class families of Atlantic salmon smolts from the USDA–ARS National Cold Water Marine Aquaculture Center (NCWMAC) in Franklin, ME (USA) were used for this study (42): 30 St. Johns River Strain (SJR) families selectively bred for growth and sea lice resistance, 5 SJR control families of neutral breeding value, and 5 Gaspe New Brunswick (GNB) randomly bred regional control families. All fish were individually tagged with passive integrated transponders (PIT tags, Biomark, Boise, ID, USA) for family origin identification and transported from the NCWMAC to the University of Maine Diagnostic Research Laboratory in Orono, ME (USA). Upon arrival, fish were randomly assigned to 21 tanks (500 L each) not exceeding a stocking density of 40 kg/m^3^ (~40–50 fish per tank). Tanks were maintained on an ultraviolet (UV)-disinfection-equipped recirculating saltwater system (15 ppt salinity; 11 ± 1 °C) held to biosafety level 3 (BSL-3) standards, with 10% water changes and monitoring of temperature and dissolved oxygen occurring daily (**Supplementary Table S1**). Fish were acclimated for 12 days and fed a 1%–2% body-weight maintenance diet (4-mm pellets) throughout acclimation and challenge. The use of experimental fish was under scientific research protocols of the University of Maine, Institutional Animal Care and Use Committee (IACUC Protocol #A2023-05-02) and complied with all relevant international animal welfare laws, guidelines, and policies.

### 2.2 Virus preparation

The Charlie Cove Back Bay ISAV (CCBB–ISAV) isolate (8, 43) was thawed from –80°C and cultured on the Atlantic salmon kidney (ASK) cell line. Viral titers were determined by endpoint dilution assay and expressed as TCID_50_ mL^-1^ using ASK cells and the Reed and Muench method (44). Titers were assessed prior to virus exposure to guide inoculum preparation and on the day of challenge to confirm the administered dose. Additional viral titer assays were performed in parallel using the Chinook salmon embryo cell line (CHSE-214). All cells were maintained following previously established protocols (43, 45).

### 2.3 Experimental challenge design

Following acclimation, Atlantic salmon from all 21 randomly assigned tanks were pooled in two 1-m^3^ Xactic containers with supplemental oxygen and anaesthetized with 50 mg L^–1^ tricaine methanesulfonate (MS-222). After anesthesia and individual family identification via PIT tag reading, four fish per family were intraperitoneally injected with CCBB–ISAV (10^4^ TCID_50_ mL^-1^) and transferred to the designated challenge tanks to act as infection shedders. The remaining 12 fish per family were distributed among the same tanks to facilitate horizontal transmission (**Supplementary Tables S2**, **S3**). Fish were allocated according to a pre-planned challenge matrix such that each tank contained one fish per family for a total of 40 fish per tank (**Supplementary Tables S2**, **S3**). Sixteen replicate tanks (T1–T16) were used to monitor family-wise morbidity/mortality. In total, 10 fish in each tank were injected with isolate CCBB–ISAV (10^4^ TCID_50_ mL^–1^) leaving the 30 additional fish as sentinel cohabitants (**Supplementary Table S2**). Terminally moribund and dead fish were removed twice daily.

To examine viral tissue loads and transcriptomic response differences between families, an additional four tanks (T17–T20) each containing 40 fish (1 fish per family) were exposed to ISAV via cohabitation of eight additional fish of mixed-family origin injected with 10^4^ TCID_50_ mL^–1^ ISAV (**Supplementary Table S2**). One of the four designated sampling tanks was sampled at 11, 21, 35, and 49 dpc. Briefly, fish were removed from the challenge tank and euthanized via MS-222 (250 mg L^–1^). Samples (~20 mg) were collected from gill, heart, spleen, and kidney and flash frozen (–80°C) individually in 1.5-mL centrifuge tubes for molecular analyses. In the event a fish originally assigned to an experimental or sampling tank lost its PIT tag during rearing or did not survive transport and acclimation, the fish was replaced with a mixed-family origin fish. Replacement was done to ensure similar cohabitation pressure and densities among mortality-monitoring (T1–T16) and sampling tanks (T17–T20). Similarly, on the final sampling day (49 dpc), fish in the designated sampling tank that had succumbed to disease during the challenge were replaced with a fish of like-family origin from tanks 1–16 if possible. Fish that had succumbed to disease in the tank designated for 35 dpc sampling could not be replaced as no alternatives were available at that time. Lastly, a sentinel control tank (T21) containing 32 mixed-family origin fish was maintained to monitor for unintended ISAV exposure via the recirculating system. Sentinel fish (eight fish per time point) were sampled in parallel with T17–T20 sampling to verify the absence of infection in system water. In all tanks, terminally moribund or dead fish were removed twice daily and at least 10% per day were subjected to clinical examination and organ collection for virus isolation and/or qPCR viral nucleic acid identification. Tissues collected from mortalities were frozen from each fish individually in 1.5-mL centrifuge tubes for molecular analysis (one tissue type per tube) and pooled for potential virus re-isolation (kidney, spleen, heart, and gill) at −80°C prior to analysis.

### 2.3 Virus detection via cell culture

Cultured cells, including ASK and Chinook salmon embryo (CHSE-214), were maintained as described in Molloy et al. (45). Frozen samples from select mortalities were thawed and processed following previously published protocols (45). Briefly, kidney tissues from sampled mortalities were diluted 1:5 (W:V) with sterile phosphate buffered saline (PBS) and gently homogenized. Homogenized tissues were then further diluted 10-fold in minimum essential medium (MEM; Invitrogen, Carlsbad, CA) or Leibovitz 15 (L-15; Invitrogen) both supplemented with gentamicin (50 µg L^–1^). A syringe filter (0.45 µm; MilliporeSigma, Burlington, MA) was used to clarify homogenates and reduce extraneous microbial contamination prior to cell inoculation (46). Inoculated wells were examined regularly for cytopathic effects for 14–21 days.

### 2.4 Virus RNA detection in tissues via RT-qPCR

Total RNA from sampled fish (kidney, spleen, gill, and heart) and select mortalities (spleen) was extracted using the MagMax Pathogen RNA/DNA kit (Applied Biosystems, Waltham, MA). The average sample weight from kidney, spleen, gill, and heart was 10.2, 9.0, 16.9, and 15.0 mg respectively. All extractions for RT-qPCR were carried out using a Kingfisher Apex Automated Extractor (Thermo Fisher Scientific, Waltham, MA) and eluted nucleic acid was quantified using a Nanodrop One (Thermo Fisher Scientific). Quantified nucleic acid was subjected to a tri-plex RT-qPCR to detect ISAV HPR0, ISAV HPRΔ, and the Atlantic salmon housekeeping gene *ef1α* as previously described (47–49). Each 10-µL reaction contained the following: 2.5 µL of UltraPlex 1-Step ToughMix (Quantabio, Beverly, MA), 700 nM each HPR0/HPRΔ primer, 350 nM HPR0/HPRΔ probe, 400 nM each HPR0 specific primer, 200 nM HPR0 specific probe, 200 µM each *ef1α* primer, 100 nM *ef1α* probe, <600 ng of nucleic acid, and nuclease-free water. Reactions were prepared and performed in 96-well plates on a CFX 1000 ThermoCycler system (Bio-Rad Laboratories, Hercules, CA). Resultant amplification data were analyzed using the CFX Maestro™ Software. Ct values for samples were calculated using a linear regression of a synthetic (Gblock) ISAV HPRΔ standard ran in duplicate spanning a dynamic range of 10^1^–10^7^ copies per reaction. Copies of ISAV segment 8 RNA per mg sample was back-calculated from initial sample weights and subsequent dilutions.

### 2.5 Virus RNA detection in water via RT-qPCR

System water samples (250 µL) were collected from all tanks on days 11, 21, 35, and 49 dpc using a pipette and flash frozen (–80°C). Viral RNA was extracted from thawed samples using 1 mL of Trizol LS (Invitrogen) and a stainless-steel 5-mm bead and homogenized in a TissueLyzer II (25 Hz, 2 min; Qiagen, Hilden, Germany). RNA was phase-separated from contaminating DNA and protein by adding 100 µL of 1-bromo-3-chloropropane, mixing via inversion, incubating at room temperature for 2 min, and centrifuging (12,000 × *g*, 15 min, 4 °C). The aqueous phase (500 µL) was removed and mixed with an equivalent volume of isopropanol supplemented with Pellet Paint^®^ colored co-precipitate (1:500; Novagen, Madison, WI) and RNA precipitated via centrifugation (16,000 × *g*, 10 min, 4 °C). The resultant pellet was washed two times with 75% ethanol, allowed to air dry for 2–5 min, and resuspended in 20 µL of nuclease-free water at 55 °C for 10 min with frequent mixing. RNA was used in the same RT-qPCR detailed above, using 2 µL of input RNA per 15-µL reaction.

### 2.6 Host gene expression analysis via digital PCR

Nucleic acid extracted from heart, kidney, and spleen samples from fish belonging to the 12 most phenotypically ISA-resistant and -susceptible families at 11 and 21 dpc was used for analysis. ISA-resistant and -susceptible fish were identified by family-based mortality as previously described (32). Days 11 and 21 post challenge were chosen to represent early and peak infection, respectively, based on viral load and mortality data. Extracted nucleic acid was subjected to DNase treatment using the Qiagen DNase I kit (Qiagen). In brief, 6 µg of RNA was added into a 1.5-mL conical tube and total volume was brought to 45 µL using nuclease-free water. Next, 5 µL of buffer RDD (Qiagen) was added and mixed, followed by 1 µL of DNase I enzyme prepared as described in the kit protocol. Following enzyme addition, reactions were gently mixed via pipetting and were incubated at 37 °C for 30 min. Following DNase treatment, samples were brought to a volume of 100 µL and cleaned using the RNeasy MinElute kit following the manufacturer’s protocol. RNA was quantified and 1 µg of DNase-treated RNA was subjected to reverse transcription using the Applied Biosystems High-Capacity cDNA Synthesis Kit (Applied Biosystems, Waltham, MA). Digital PCR (dPCR) was completed using the QIACuity dPCR system with 8.5k 96-well plates. The 12-µL reactions consisted of 4 µL of QIACuity EvaGreen Master Mix, 400 nM each primer, 1–4 µL of cDNA, and nuclease-free water. Prior to moving to dPCR, primer pairs were screened for efficiency and specificity via qPCR in a five-step fourfold serial dilution of pooled spleen RNA. A list of all primers can be found in **Supplementary Table 4**. An unpaired, non-parametric Mann–Whitney test was used to compare resistant and susceptible phenotypes at each time point per target, and geometric means of multiple (2, 3) genes were compared similarly to evaluate pathway-specific regulation.

### 2.7 Host spleen transcriptomic profiling via RNA sequencing

RNA was purified from 38 select spleen samples (~10 mg each) using TRI Reagent (Thermo Fisher Scientific) following the manufacturer’s recommendations similar to Trizol LS RNA purifications from water samples described in Section 2.6. Purified RNA was then subjected to a DNase I treatment and cleaned using the Qiagen Min-elute spin columns following the manufacturer’s instructions and eluted in 20 µL of water. RNA concentration was quantified using a Nanodrop (Thermo Fisher Scientific) and 100 ng was submitted to the Oklahoma Medical Research Foundation Genomics Core (Oklahoma City, OK) for RNA sequencing (RNA-seq). RNA libraries were prepared using a Watchmaker mRNA library kit and 150-bp paired-end sequencing was completed using an Illumina NovaSeq X. Raw sequencing data were transferred to the USDA SCINet High-Powered Computing cluster, where raw FASTQ reads were trimmed using TrimGalore (0.6.6) and mapped to *Salmo salar* reference genome GCF_905237065.1 using HISAT2 (2.2.1). Libraries with less than 10 million reads or less than 70% of reads mapping to reference genome were excluded from further analysis. Libraries were sorted and indexed with Samtools (1.17), transcripts were assembled, abundance estimations were performed with StringTie (2.2.0), and differential gene expression (DGE) analysis was conducted using the R package DESeq2 (1.50.2) in R (4.5.1) comparing susceptible and resistant individuals grouped independently by time point. A false-discovery rate *p*-value cutoff of 0.05 with a 1.5-fold (0.5 log2) expression change was used as a minimum threshold for identifying differentially expressed genes. To further improve gene annotation yields, all unannotated putative protein-coding transcripts were NCBI blasted against the UniProt *Salmo salar* proteome (ID: UP000087266). Functional enrichment analysis of annotated up- and downregulated genes was completed using the function clusterProfiler::enrichGo() (4.18.1) using default parameters and the *Salmo salar* AH119615 database from Annotation Hub (Bioconductor 3.22). All codes and steps performed during RNA-seq analysis can be found on Github repository (https://github.com/polinskilab/rna-seq-analysis-ISAV-challenge-manuscript.git).

## 3 Results

### 3.1 ISAV caused clinical disease and mortality via injection and cohabitation

More than half (56%) of Atlantic salmon in T1–T16 became terminally moribund or died during the 49-day ISAV challenge in this study. Clinical signs of disease were observed in all examined mortalities (**Supplementary Table S5**). Most prevalent were general visceral hemorrhaging, splenomegaly, ascites, and intestinal edema presenting in 75%, 78%, 76%, and 73% of examined morbidities/mortalities, respectively. Virus re-isolation using cell culture was attempted from 126 of the 358 (35%) terminally moribund or dead fish following challenge (T1–T16). Cytopathic effects were observed in 56% (71/126) of cell cultures (ASK or CHSE-214) from frozen tissue pools (one tissue pool per fish), and the presence of ISAV RNA was detected in spleen tissues from all samples tested by RT-qPCR (158/158; **Supplementary Table S5**).

### 3.2 Cumulative mortality was not impacted by exposure route

**Figure 1:**
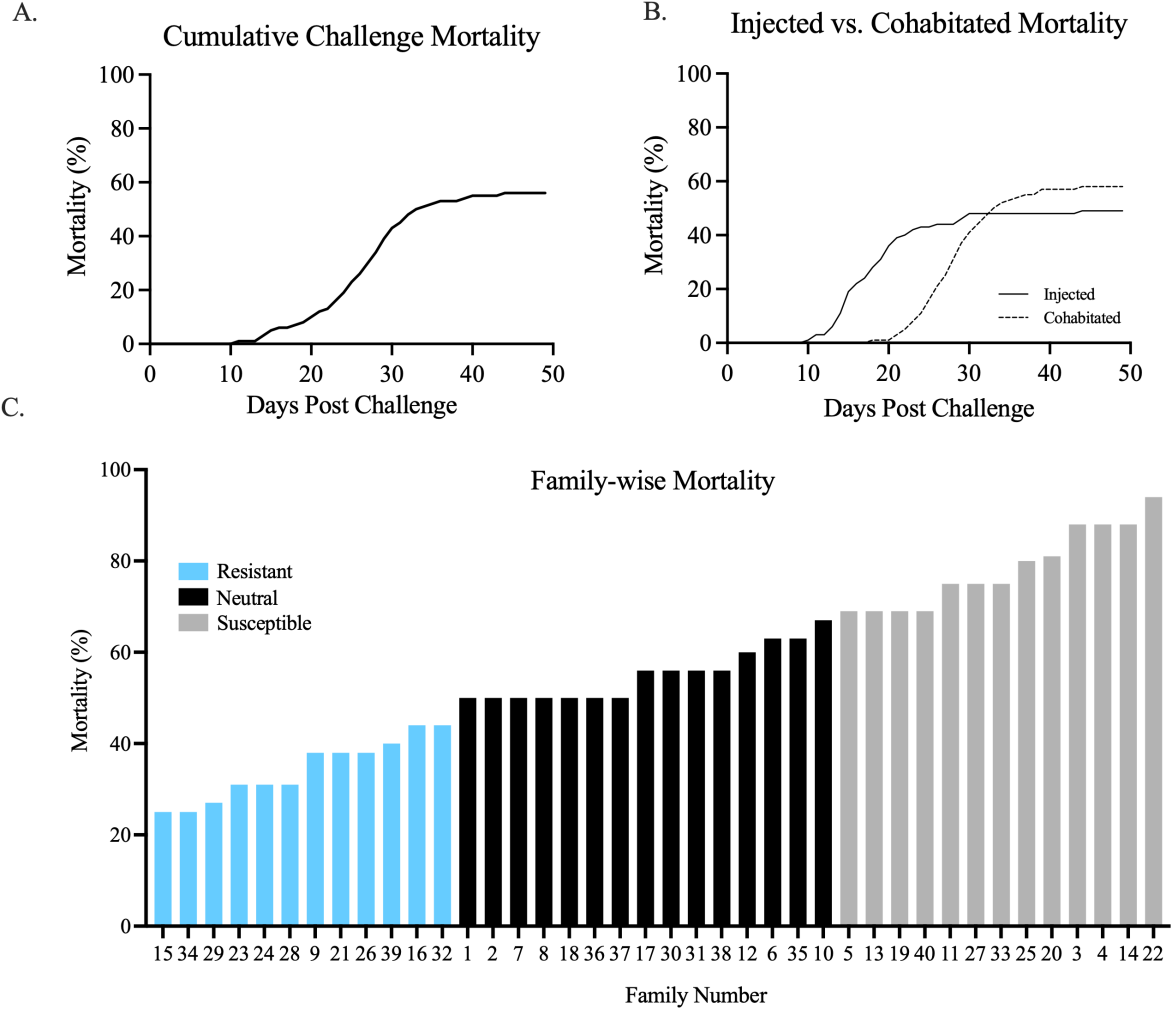
**(A)** Mortality of the 49-day experimental exposure trial. **(B)** Mortality curves comparing fish exposed via injection (solid line) vs. fish exposed via cohabitation (dotted line). **(C)** Cumulative mortality (%) of each Atlantic salmon family challenged. Families 1–10 are randomly bred controls, and families 11–40 are selectively bred. Families grouped as resistant to ISA (blue) are distinguished from families grouped as susceptible to ISAV (gray) or not assigned an experimental group (black).

Mortality in T1–T16 reached 56% by the end of the 49-day challenge experiment with very little mortality occurring in the last 10 days, suggesting that surviving fish were in the resolution phase of the disease outbreak (**Figure 1A**). Injected and cohabitated fish had similar cumulative mortality (48.4% and 58.0%, respectively; *p* = 0.65 by Log Rank survival analysis); although mortality trends were shifted approximately 10 days earlier in injected (10–30 dpc) relative to cohabitant fish (20–40 dpc; *p* = 0.003 by the Grehan–Breslow–Wilcoxon test) (**Figure 1B**). Thus, while the exposure route had an effect on disease timing, it did not affect outcome and cohabitated and injected fish were treated cumulatively for family-wise endpoint mortality analyses that identified 25%–94% family-specific mortality across the 40 families studied (**Figure 1C**).

### 3.3 NCWMAC selective breeding for growth and sea lice resistance has not incidentally impacted ISA resistance

**Figure 2:**
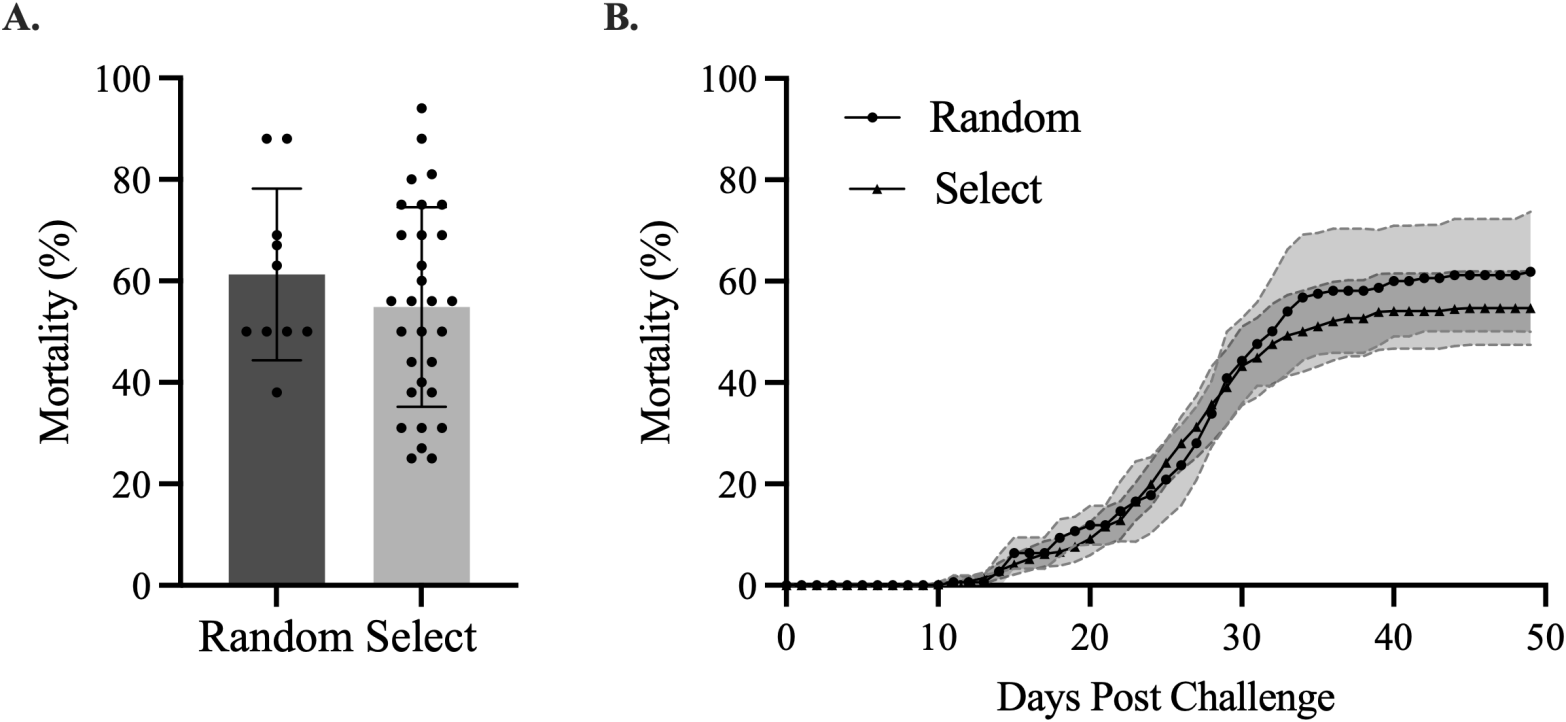
**(A)** A *t*-test comparison of the cumulative percent mortalities of randomly bred vs. selectively bred families (*p* = 0.3610). **(B)** Mortality curve for all randomly (*n* = 10) and selectively bred (*n* = 30) families. Error bars represent the 95% confidence interval.

Cumulative mortality between families bred for high growth and sea lice resistance was similar to non-selected control families (mean 55% ± 19 SD and 62% ± 16 SD; *p* = 0.6584 by Welch’s *t*-test; **Figure 2A**). Probabilities for mortality were also similar between selected and randomly bred families throughout the challenge period (*p* = 0.3624 by the Grehan–Breslow–Wilcoxon test; **Figure 2B**).

### 3.4 Large variations in family-specific ISA resistance supported ISA-resistant and -susceptible phenotype comparisons

**Figure 3:**
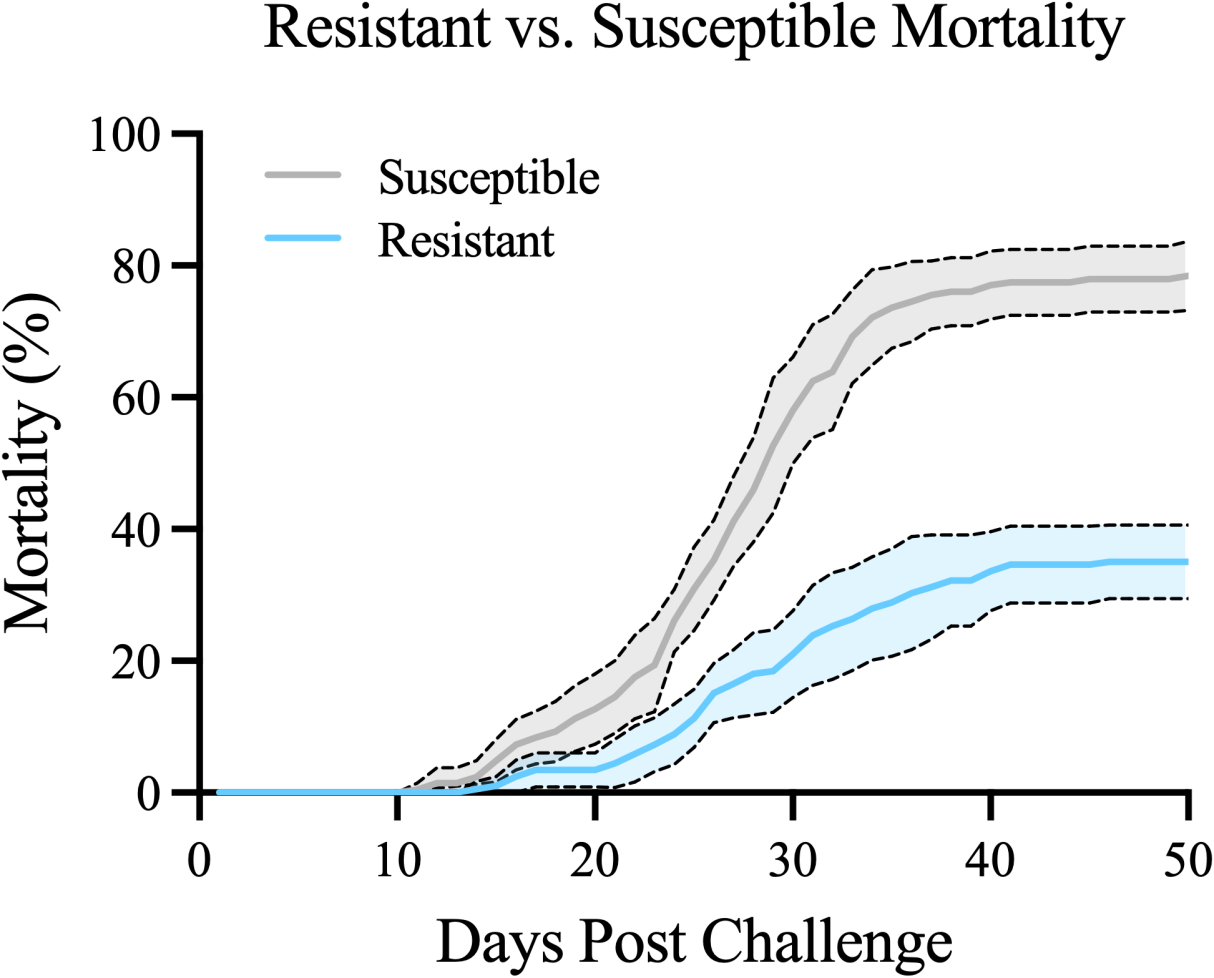
Cumulative percent mortality of the most phenotypically resistant (*n* = 12) and phenotypically susceptible (*n* = 13) families. Error bars represent 95% confidence interval from 0 to 49 dpc. *p* < 0.0001 via Welch’s *t*-test.

The large variation in family-specific mortality (25%–94%) across the 40 families studied provided an opportunity to compare characteristics between the apparently most susceptible and resistant families. This was accomplished by grouping the upper and lower 30% of families by cumulative mortality into “resistant” and “susceptible” groups, respectively (*n* = 12 and 13 families/group; **Figure 1C**). Mortality of the two phenotypes was significantly different via Welch’s *t*-test (*p* < 0.0001) (**Figure 3**). Additionally, mean mortality probabilities were more than threefold higher in susceptible vs. resistant groups (*p* < 0.0001 Log-Rank Mantel–Cox test; hazard ratio = 3.348; **Figure 3**).

### 3.5 High systemic ISAV RNA loads occurred in all organs screened with prolonged elevation in the spleen

**Figure 4:**
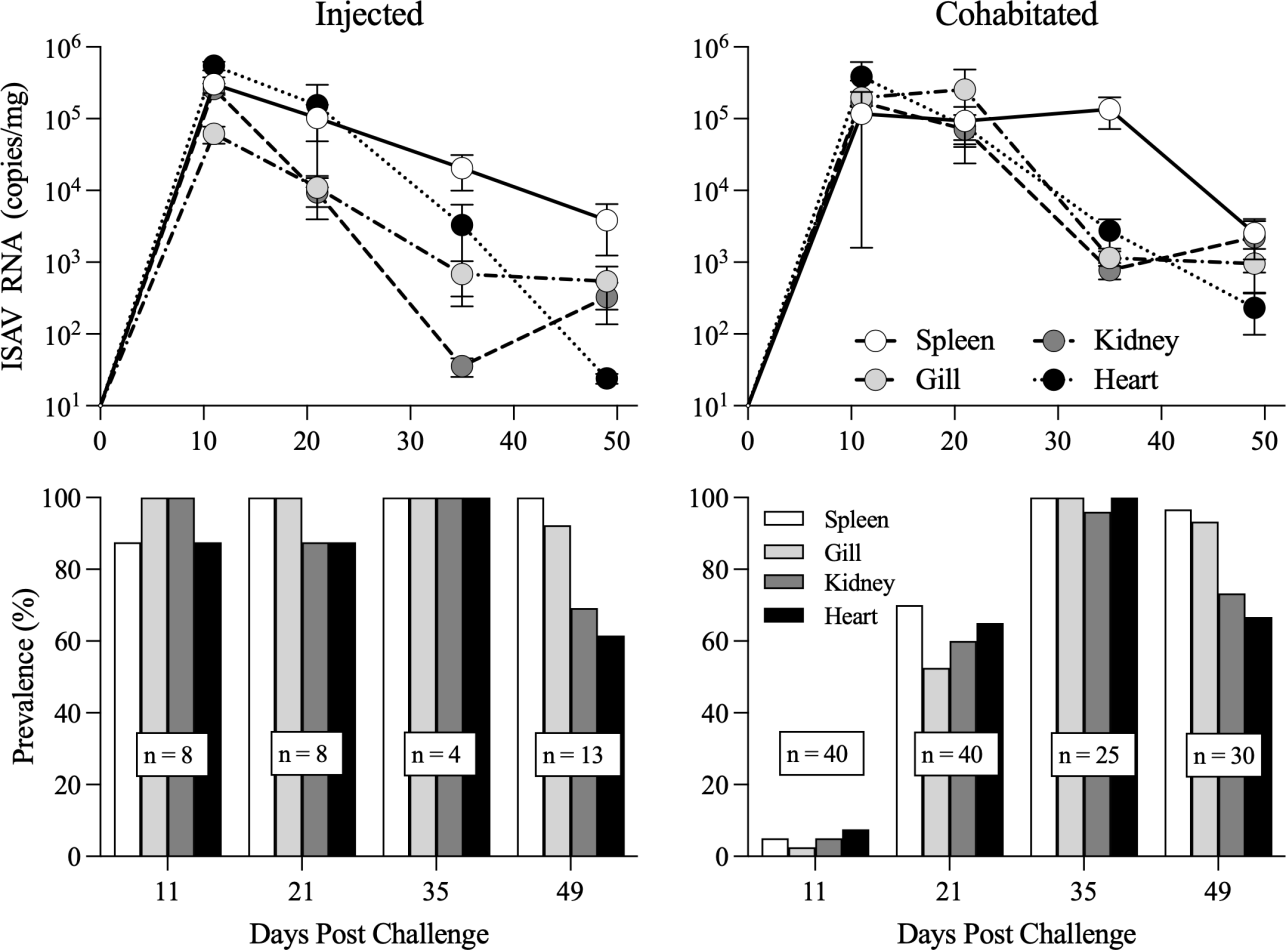
ISAV RNA loads (mean ± SEM copies; upper panels) and population prevalence (percent; lower panels) in spleen, kidney, gill, and heart samples measured at 11, 21, 35, and 49 days post challenge in both i.p. injected (left panels) and cohabitated (right panels) Atlantic salmon smolts.

After 11 days of cohabitation with ISAV-injected shedders, ISAV RNA was detected at high loads in gill, kidney, spleen, and heart of all injected shedder fish but only at low prevalence in cohabitant fish, albeit at high quantities when detected (**Figure 4**, **Supplementary Table S6**). By 21 dpc, both prevalence and loads had reached high levels (~10^4–^10^5^ copies mg^–1^ in injected fish, ~10^5^ copies mg^–1^ in cohabitated fish), where similar viral loads were detected across the four organs tested (gill, kidney, spleen, and heart). By 35 dpc, ISAV RNA loads began to decline; however, markedly higher quantities were detected in spleens of infected fish than in any of the three other organs screened. At challenge termination (49 dpc), viral loads had dropped substantially and were again similar across all four organs screened (**Figure 4**).

### 3.6 Viral infection dynamics were similar between phenotypically resistant and susceptible salmon

**Figure 5:**
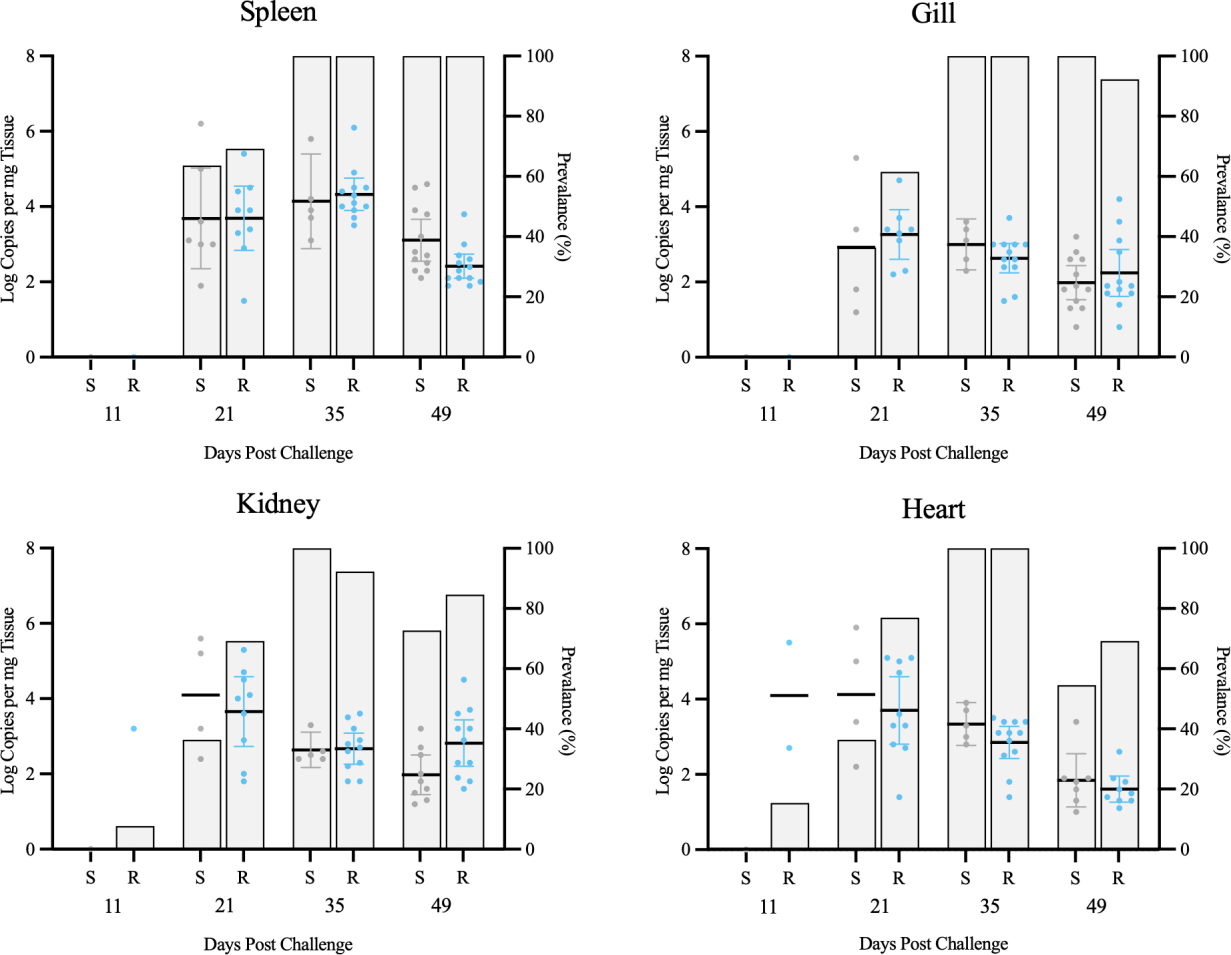
Mean (line) ± 95% confidence intervals (error bars) of ISAV RNA and infection prevalence (bars) per organ type across all four time points. R: Resistant, S: Susceptible. The black line represents the mean value and error bars represent the 95% confidence interval where the number of samples is >4. Individual values are represented with dots and correspond with the left Y-Axis. Right *Y*-axis is a measure of percent infection prevalence (i.e., number of ISAV-positive samples divided by total number of samples tested) and is depicted with bars.

Despite the substantial (3.348 hazard ratio) difference in likelihood for mortality in resistant vs. susceptible families, ISAV loads and prevalence were highly similar within organs of salmon from both sets of families (**Figure 5**). Indeed, during the entire course of infection, ISAV genomic RNA loads were similar in spleen, kidney, heart, and/or gill when comparing fish from resistant and susceptible families (*p* > 0.111), with slight differences only observed in the spleen and kidney at 49 dpc where resistant fish had lower ISAV RNA loads in the spleen (3.108 ± 0.879 vs. 2.415 ± 0.532 log copies per milligram; *p* = 0.030) but higher loads in the kidney (1.978 ± 0.685 vs. 2.818 ± 0.917 log copies per milligram; *p* = 0.031). Infection prevalence also followed similar trends in both resistant and susceptible families (**Figure 5**), indicating that viral RNA loads were similar when detected and detection prevalence was consistent across all organ and time points between phenotypes, highlighting the systemic nature of ISAV RNA distribution regardless of phenotypic ISA resistance.

### 3.7 ISAV RNA was detected ubiquitously in culture water during peak to late-stage infections

**Figure 6:**
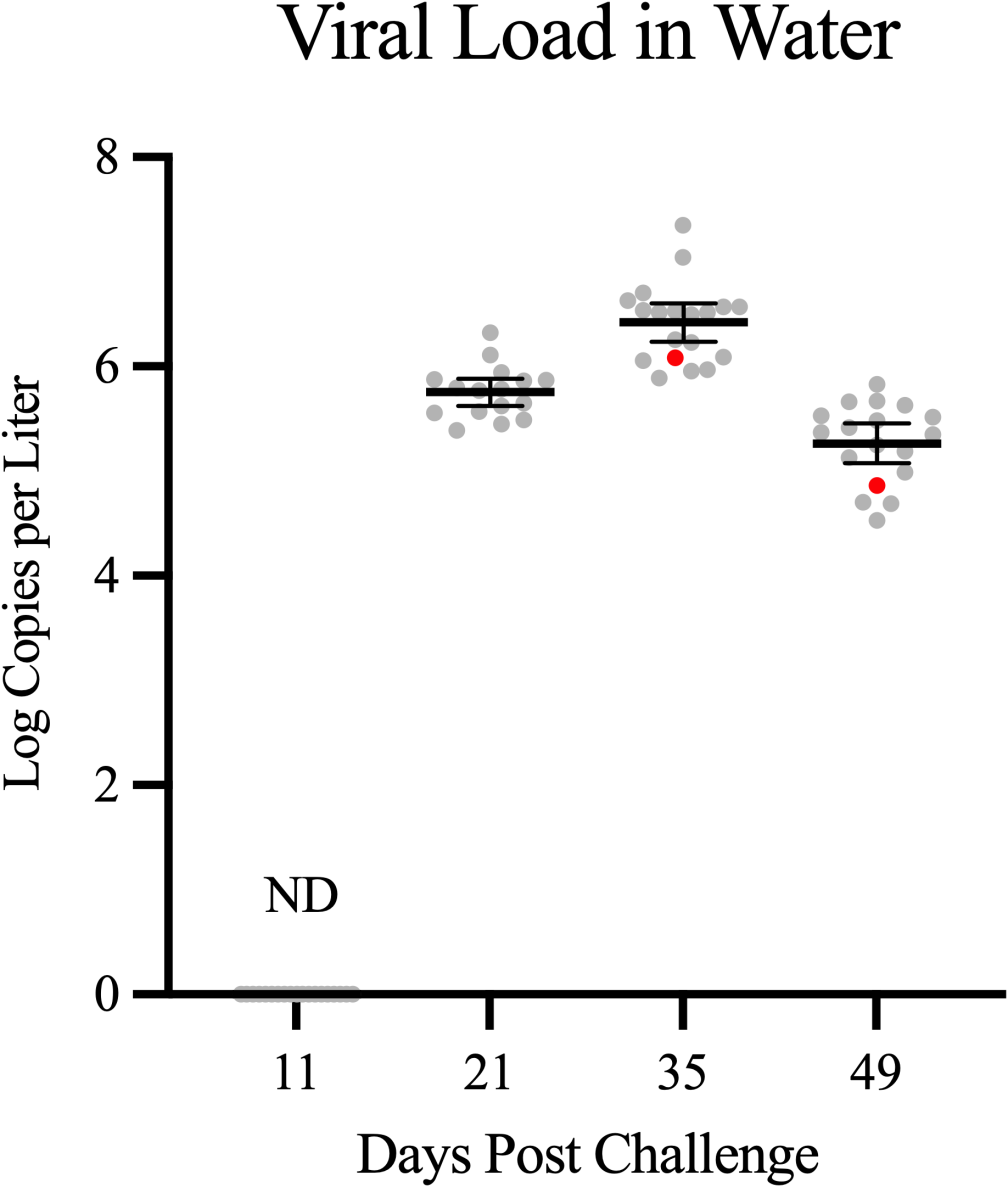
Mean ( ± 95% CI) copies of ISAV RNA per liter system water from all 17 non-sampled experimental tanks. Water collected from the sentinel control tank (red) is distinguished from water from challenge tanks (gray). ND, not detectable.

ISAV RNA was not detectable in any tank water screened during early infection and transmission (11 dpc). However, by 21 dpc—when mortality was beginning to peak—ISAV RNA was detected in water from 14 of 16 challenge tanks tested where ISAV RNA ranged from 2.5 to 20.9 × 10^5^ copies L^–1^ of tank water (**Figure 6**, **Supplementary Table S7**). Viral RNA was not detected in the sentinel tank at this time point. By 35 dpc—when peak mortality was coming to an end—ISAV RNA was detected at high quantities in water of all challenge tanks as well as in the sentinel tank, with copies per liter ranging from 7.8 to 223.9 × 10^5^. By the termination of this study (49 dpc), virus RNA was still detectable in water of all tanks tested but at generally lower quantities (0.3–6.8 × 10^5^ copies L^–1^) than at 35 dpc. Despite detection in the sentinel tank at both 35 and 49 dpc, viral RNA could not be detected in spleen, gill, kidney, or heart tissues of any sentinel fish tested at any time point sampled (*n* = 8 per time point), suggesting that ISAV RNA in water was not indicative of infectious virus particles of sufficient quantity to initiate systemic infections of naïve fish.

### 3.8 RNA sequencing of spleen during early infection identifies differential expression patterns in resistant vs. susceptible families

Spleen was chosen for RNA-seq analysis due to its representation of high systemic ISAV transcripts and the prolonged maintenance of high ISAV RNA loads relative to other organs during the later stage of infection (**Figure 4**), suggesting it may be a significant site for viral replication and/or immune responsiveness. Emphasis was further placed on early time points because (i) current knowledge of ISAV disease suggests that effective resistance must be mounted quickly (16, 27), and (ii) these time points allowed access to larger sample sizes as they occurred before the majority of morbidity/mortality that reduced access to experimental animals, particularly those from susceptible families. Thus, of the 38 initial samples submitted for sequencing, 32 samples produced libraries above the 10 million read per library cutoff with greater than 70% of reads mapping to the reference genome: 12 on day 11 (3 resistant, 4 susceptible, and 5 controls), 7 on day 21 (4 resistant and 3 susceptible), and 8 on day 49 post-challenge (4 resistant and 4 susceptible). Mean read count per retained library was 14,058,676 (95% CI 13,347,133–14,770,220; *n* = 32), with 83.1% (95% CI 82.1%–84.2%; *n* = 32) mapping to the Atlantic salmon genome.

**Figure 7:**
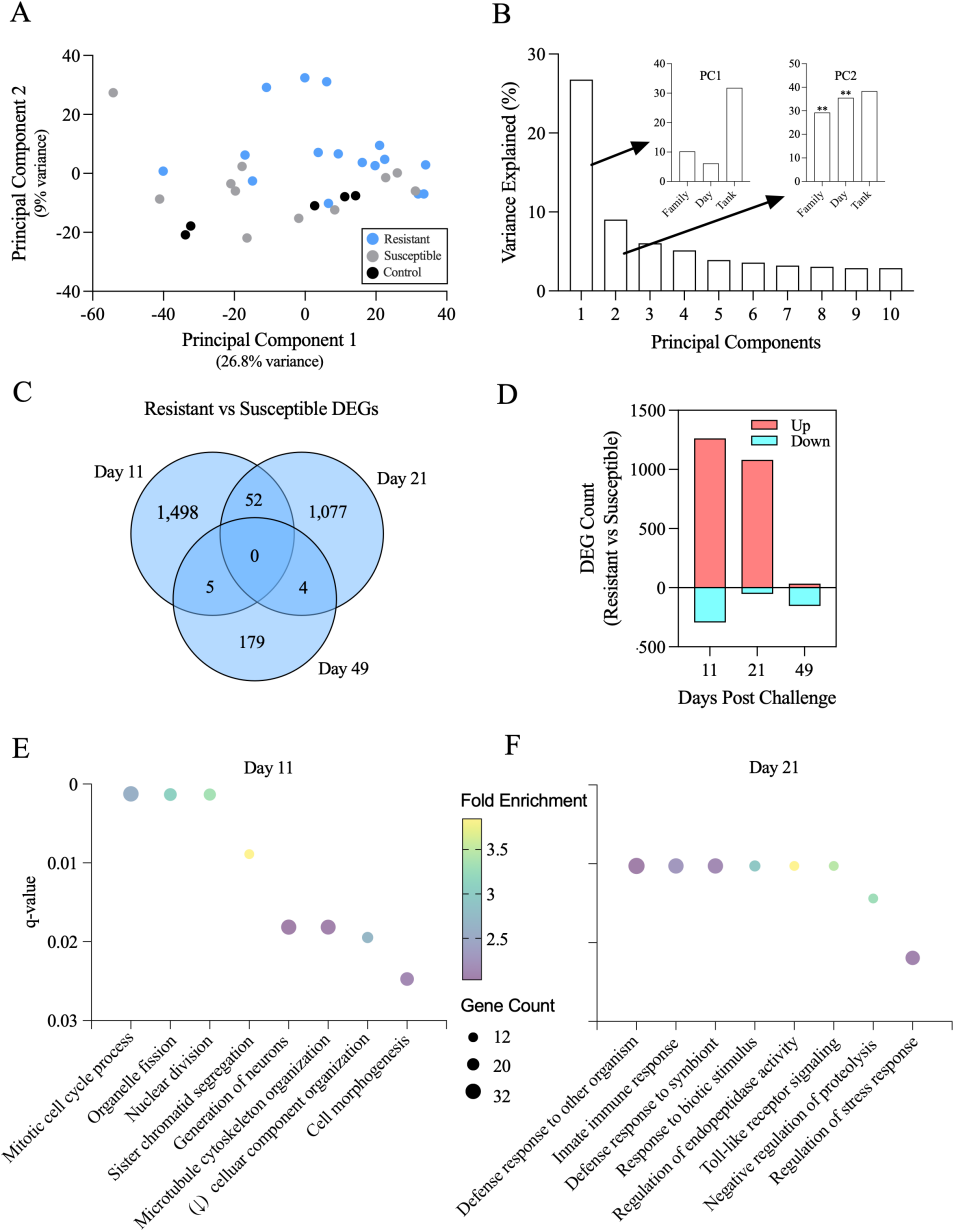
**(A)** Exploration of variance of Atlantic salmon spleen RNA-seq data using principal component analysis. Component scores grouped by ISAV phenotype (resistant and susceptible) relative to unchallenged controls are visualized via the two principal dimensions of variance. **(B)** The contribution to total variance associated with each of the top 10 principal dimensions (main panel) with specific contributions from three putative predictor variables (family, sample day, and culture tank) to the total variance within the first two principal dimensions (inserts). ** indicates *p* < 0.01 by integrated ANOVA and Benjamini–Hochberg false discovery rate adjustment. **(C)** Venn diagrams present the number of discrete and shared differentially expressed (both up- and downregulated; 0.05 FDR-adjusted *p* < 0.05 with greater than 1.5-fold change) genes identified from spleen RNA-seq data relative to time point of collection as compared between individuals from resistant vs. susceptible families. **(D)** Gene counts (0.05 FDR-adjusted *p* < 0.05 with greater than twofold change) associated as being upregulated (red) or downregulated (blue) in individuals from resistant families relative to susceptible families. **(E, F)** Function pathway enrichment assessment for differentially expressed genes as determined at either 11 **(E)** or 21 **(F)** dpc in individuals from ISAV-resistant relative to -susceptible families.

Initial data exploration was conducted via principal component analysis using the DESeq2 package (**Figure 7A**). Although the major source of variance (Principal Component 1, ~29% total variance) between libraries was not clearly attributable to defined experiment variables (**Figure 7B**), the second strongest source for variance (Principal Component 2, ~9% total variance) was influenced strongly by both family and day of sampling (**Figure 7B**). Further analysis revealed that more than 1,100 genes at both 11 and 21 dpc were highly differentially expressed (>1.5-fold change; *p* < 0.05) between resistant and susceptible families (**Figure 7C**), for which most were upregulated in the resistant phenotype (**Figure 7D**). By day 49 post-challenge, differences in transcriptomic profiles appeared less clear, possibly due to similar responsiveness and/or limited sample numbers at this later time point.

Pathway enrichment analysis for annotated differentially expressed genes further identified active cell proliferation via mitotic signaling pathways as being upregulated in resistant vs. susceptible fish at 11 dpc (**Figure 7E**). By 21 dpc, the most significant differences between resistant and susceptible fish appeared to relate to a more robust direct immune defense associated with resistance (**Figure 7F**). All RNA-seq analysis code and resulting data are available at the GitHub repository: https://github.com/polinskilab/rna-seq-analysis-ISAV-challenge-manuscript.

### 3.9 Targeted gene expression assays highlighted early viral recognition in kidney as important in disease resistance

**Figure 8:**
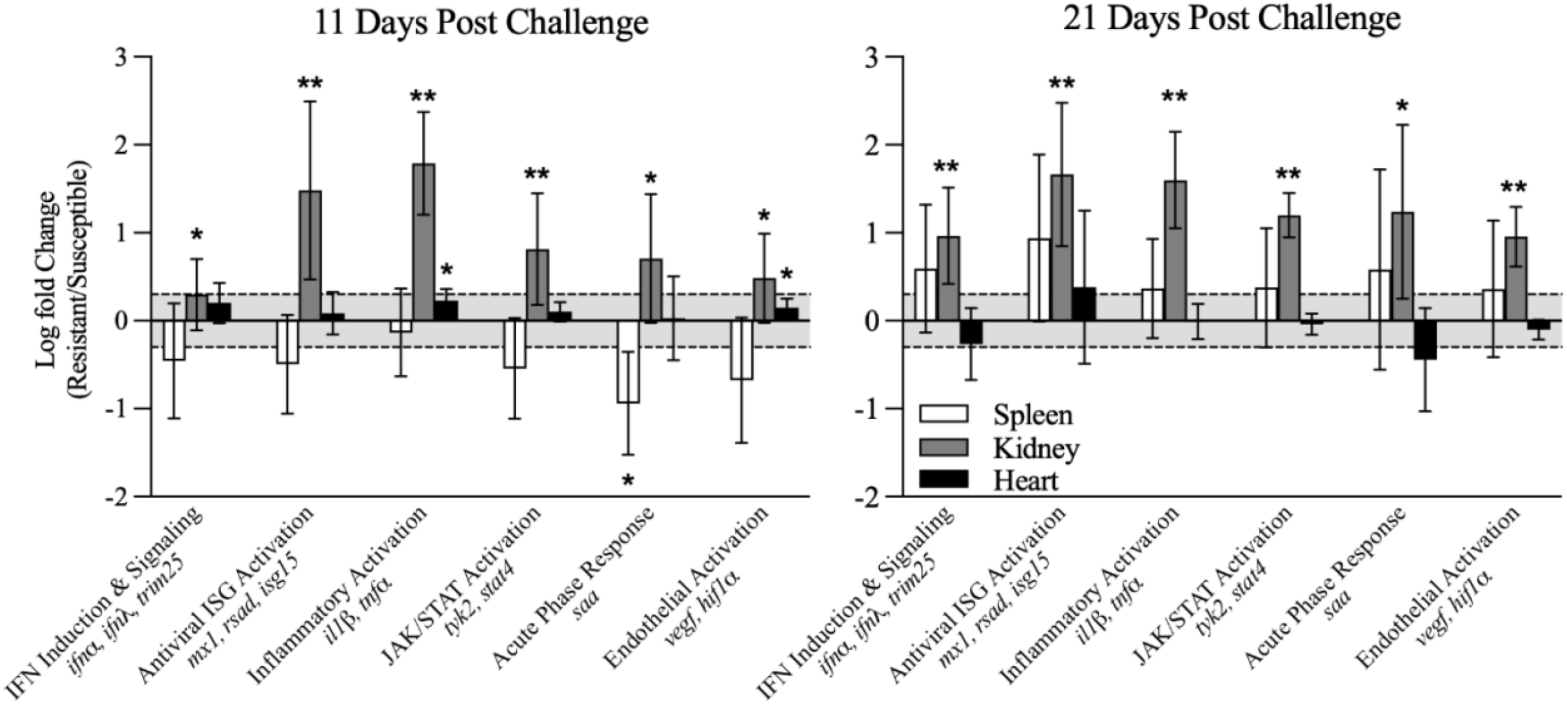
Mean log-fold change ( ± 95% CI) derived from geometric means (copies/µg total RNA) of screened genes grouped based on functional pathway involvement. The shadowed area represents ±2-fold change suggested as a minimum threshold for biological relevance. All gene expression data are available in **Supplementary Table S6**.

Transcriptomic differences identified in the spleens of resistant and susceptible families above coupled with the systemic distribution of virus suggested that responsiveness in other organs may contribute synergistically or separately to a disease resistance phenotype. We therefore conducted targeted gene expression analyses in the spleen, kidney, and heart to identify potential differential activation of pathways likely triggered by ISAV infection and subsequent ISA disease. Specifically, we investigated interferon (IFN) induction and signaling measured by *ifnα*, *ifnγ*, and *trim25* transcripts; antiviral interferon stimulated gene (ISG) activation via *mx1*, *rsad2*, and *isg15* transcripts; inflammatory activation via *il1β* and *tnfα* transcripts; JAK/STAT (Janus kinase/Signal Transducer and Activator of Transcription) pathway activation via *tyk2* and *stat4* transcripts; acute phase response activation via *saa* transcripts; and endothelial growth activation via *vegf* and *hif1α* transcripts. This identified that early kidney responsiveness was the major differentiator between resistant and susceptible families. All pathways assessed showed elevated activation in resistant fish kidney, particularly the pathways involved in antiviral ISG activation and inflammation (**Figure 8**), relative to susceptible individuals. All gene expression data are available in **Supplementary Table S6**.

## 4 Discussion

### 4.1 Selective breeding efforts have not affected ISA resistance, but family-associated differences remain

An initial aim of this study was to determine if the NCWMAC Breeding Program had incidentally impacted ISAV resistance via more than four generations of selective breeding for growth and sea lice resistance that has allowed for a potential doubling of harvest weight over a typical production cycle (NCWMAC, unpublished data, 42). Rapid host growth rates coupled with high muscle mass can be leveraged in favor of viral replication (50), such as with Marek’s disease in poultry (51) and Swine fever in pigs (52). In this instance, however, growth selection in conjunction with sea lice resistance did not appear to impart a negative impact for resistance to ISA or ISAV by salmon in this study, although the power to identify subtle effects was limited using only 40 families (30 selected and 10 control; SD ~16%–19%). Nevertheless, these findings provide a tentative example of sustainability in implementing a multi-trait selection program (growth + parasite resistance) without apparent compromise in viral resistance.

A second aim of this study was to confirm family-based phenotypic variation within our breeding population. In a recent study, Gervais et al. (33) assessed ISA resistance in 194 families of European stock Atlantic salmon (~15 fish per family) and identified large variations in family-specific resistance (7%–100% linear trend of family-specific mortality) with a polygenic architecture of resistance distributed across several chromosomes. We did not examine genetic architecture for resistance in this study; however, we did identify a highly comparable variance structure for family-wise resistance within our 40-family cohort of North American Atlantic salmon (25%–94% linear trend of family-specific mortality), confirming similar family-wise resistance patterns against ISA in both North American and European Atlantic salmon stocks.

### 4.2 ISA resistance does not equal ISAV replication resistance

A common mechanism in the host cellular antiviral immune response is the direct suppression of viral replication and is exemplified by influenza A virus, a relative to ISAV in the family *Orthomyxoviridae* (53). To that end, our initial hypothesis was that phenotypic ISA (i.e., disease) resistance would be associated with a decrease in ISAV replication within the host. An interesting finding herein was that viral load data (as measured by viral genomic transcripts) were statistically similar at all time points and in all organs (i.e., gill, heart, kidney, and spleen) between disease-resistant and -susceptible phenotypes. This suggests that the innate effectiveness for an Atlantic salmon to mitigate and control tissue damage (i.e., disease) does not require successful suppression of ISAV transcription and likely replication. This does not necessarily mean that blocking downstream viral processes such as virion assembly, virion cellular egress, or viral sequestration are not important for limiting disease severity—these were beyond the scope of this study to measure. It does, however, provide a clear example that tolerance to viral transcription in favor of tissue-level control of pathology can be an effective approach for minimizing morbidity following systemic infection by at least one isolate of ISAV. This is further supported in another orthomyxovirus, influenza A virus, where viral load appears generally decoupled from pathology in mammalian systems (54, 55). Nevertheless, previous research focused on Tilapia lake virus (TiLV)—which belongs to a closely related viral family (*Ammoonviridae*)—found disease resistance to be synonymous with decreased viral loads (56). We speculate that this may be due to differences in cellular/tissue tropism between ISAV and TiLV and/or differences in direct cytotoxicity. Whatever the case, it highlights considerable mechanistic diversity for generating disease within fish orthomyxo-like viruses.

### 4.3 Systemic viral loads and timing suggest organ-specific roles throughout ISAV infection

For salmon exposed via cohabitation in this study, ISAV infections became highly systemic within the first 3 weeks post-challenge, as indicated by near-complete prevalence across gill, kidney, spleen, and heart at 21 dpc where viral RNA loads during this period were uniformly high (**Figure 4**). This provides indirect evidence for active replication in multiple organs. By 35 dpc, a striking divergence emerged: while prevalence remained at 100% in all organs, viral loads declined sharply in gill, kidney, and heart by approximately two orders of magnitude, yet persisted at high levels in spleen. This suggests a unique role for the spleen during late-phase ISAV infection as a site of prolonged viral replication and/or delayed clearance. Specifically, we hypothesize that splenic ellipsoids and melano-macrophage centers may be specifically accumulating viral particles and substrates via the filtration capacity of this organ. This is supported by a previous study identifying that salmon splenic filtration structures respond early to ISAV infection via congestion and melano-macrophage proliferation (2) and are an expected site to accumulate high load systemic viral particles like ISAV (57), and the high vascularization of this tissue favors ISAV filtration over avascularized interbranchial lymphoid tissue (58).

We also observed key differences in viral load dynamics associated with exposure route, despite not finding overall differences in clinical outcome. Austbo et al. provided evidence of the gills being an early hub for ISAV replication indicated by high viral loads of total and replicating ISAV at ~48 h post bath exposure, suggesting a critical role as an initial entry point (59). While we did not examine time points earlier than 11 dpc, we observed viral loads in the gill to be 1–1.5 magnitudes higher in cohabitation-exposed fish at 11 and 21 dpc compared to injection-exposed fish. Furthermore, the gill presented significantly lower viral loads than all other tissues in injected fish at 11 dpc, while viral loads remained similar to or above that of other tissues in cohabitation-exposed fish during peak infection points (11 and 21 dpc). These findings provide further support for gill being strongly involved in early replication and dissemination for ISAV.

By 49 dpc, viral RNA declined substantially across all organs with the exception of the kidney, but prevalence remained highest in spleen (100%) and gill (92%), indicating incomplete clearance and a potential for continued shedding and carrier status. These combined patterns highlight (1) gill as a likely portal of entry with slow clearance and a long-lasting potential shedding source, (2) kidney and heart as sites of efficient viral clearance relative to other organs, and (3) the spleen as a probable reservoir for persistent viral RNA. Thus, our results suggest spleen as the most reliable tissue for late-stage ISAV diagnostic targeting while screening for subclinical and/or carrier status could incorporate both spleen and gill.

Integration of both tissue and water data from this study reveals a clear link between systemic infection and environmental shedding. While ISAV RNA was undetectable in water at 11 dpc, mean concentrations rose sharply by 21 dpc and peaked at 35 dpc, coinciding with near-complete organ prevalence and persistent high viral loads in spleen. By 49 dpc, water RNA declined but was still readily detectible in all tanks, potentially supporting incomplete clearance and carrier status in surviving fish. Interestingly, however, similar detection prevalence for viral RNA in water of the sentinel unchallenged control tank—where ISAV was never detected within fish tissues—highlights that water-associated “eDNA” in this instance was not representative of circulating infectious virus. Coupling this with previous observations for environmental stability of ISAV in seawater to be low (a matter of hours; 60), our eDNA detections indicate a prolonged persistence of ISAV RNA in water well past the period for an ISAV particle being infectious. If and how this persistence may occur in fish tissues is unknown but would be worthy of further investigation—particularly in defining the role of the spleen in maintaining persistent ISAV RNA loads.

### 4.4 The kidney is an early, dynamic antiviral responder in resistant phenotypes

The kidney functions as the primary lymphoid organ in bony fish, with the head and trunk kidney both exhibiting immune activity, though the head kidney is recognized as the prominent site for hematopoiesis (61). Herein, we found the most pronounced differences between resistant and susceptible fish via gene expression in the kidney. Prior work by Gervais et al. (39) similarly identified the head kidney as a major responder during ISAV infection, with strong induction of antiviral pathways—including interferon and NLR-associated signaling pathways—in virus-exposed fish relative to controls (39). Interestingly, minimal differences in transcriptomic profiles were found between resistant and susceptible fish at 0 and 14 days, suggesting that key differential responses happened near 7 dpc (39). In contrast, we observed significant resistance-associated differences in kidney gene expression at both 11 and 21 dpc, indicating that differential kidney responsiveness persists beyond early infection in our system. While the timing of sampling differs between studies, together these findings reinforce the kidney as a central organ for mounting resistance-associated responses to ISAV infection. Though not explored in the context of disease resistance, early antiviral kidney responses have been identified in other fish species infected with RNA viruses including viral hemorrhagic septicemia virus in flounder (62) and infectious pancreas necrosis virus in cultured salmon head kidney (SHK-1) cells (63).

The early response of the kidney (11 dpc) included significant antiviral ISG and inflammatory marker transcripts in resistant fish compared to susceptible ones. Interestingly, upregulation of screened ISGs did not coincide with upregulation of IFNs and IFN inducers, and we hypothesize that an early IFN spike may have been missed in sampling between 0 and 11 dpc. Further supporting this theory, Gervais et al. found significant upregulation of *ifnα/β* receptor transcripts in ISAV-infected fish at 7 dpc, with ISGs and IRFs more significantly upregulated by 14 dpc (39). A rapid (<96 h) IFN response to ISAV has also been identified *in vitro* in cultured TO cells derived from ASK and exposed to virus (64). By 21 dpc in the present study, we did, however, begin to see an increase in *ifnα*, *ifnγ*, and *trim25* transcripts, and a slightly more substantial increase in ISG activation transcripts. The cycling of IFNs and ISGs is interesting, as IFNs function in an auto- or paracrine capacity, with secreted IFNs ultimately leading to the secretion and generation of more IFNs and ISGs. One possibility is that an initial, pre-11-dpc IFN induction in the kidney leads to an early, but sustained, increase in ISG transcripts, and during peak infection, a secondary IFN spike occurs systemically (kidney and heart) that helps in resolving infections and/or disease. Evidence of a later (21 dpc) antiviral gene spike in the heart was also shown in Lauscher et al. (65). Alternatively, the minimal log-fold change between resistant and susceptible fish could suggest that resistant and susceptible fish manifest a similar initial IFN response, but resistant fish maintain IFN induction longer or is less efficiently blockaded by viral components as is evident through subsequent ISG and pro-inflammatory cytokine activation.

Compared to other organs, the kidney also saw significant upregulation of two genes associated with the JAK/STAT pathway in resistant fish compared to susceptible ones (**Figure 8**). The JAK/STAT pathway serves as a liaison between the extra- and intracellular environment (66) and specifically recognizing extracellular IFN and triggering ISG production (67). In the kidney, we saw resistant fish upregulating ISGs, pro-inflammatory cytokines, and JAK/STAT pathway-associated targets compared to susceptible fish at both time points synergistically (11 and 21 dpc), strongly indicating that host viral recognition and deployment of antiviral responses are enhanced in resistant fish at these time points. Specifically, the increased transcription of *stat4* and *tyk2* suggests that resistant fish have enhanced T-helper-1-like cell-mediated immune responses for combating viral invasion relative to their susceptible counterparts.

Rapid recognition of ISAV in resistant fish confers a timely advantage in establishing an antiviral state that may manifest in one or more beneficial outcomes: limiting viral replication and burden before they overwhelm the immune system, preventing excessive or prolonged inflammation and tissue damage, and/or promoting effective downstream adaptive immune responses (68, 69). In this study, we did not observe direct evidence for preventing viral replication and thus conclude that resistance in this specific instance is being driven by either enhanced tissue damage mitigation or adaptive responses and not by blocking infection at the portal of entry. As adaptive responses are generally slow to initiate in cold water fish (70), we speculate that the earlier and more robust antiviral state established in ISA-resistant fish is supporting improved tissue damage mitigation, perhaps through reduced viral egress, direct cell lysis, or improved wound healing.

These data cumulatively highlight that rapid recognition with the central hematopoietic/immune cell-generating organ of the kidney is a key differentiator for innate resistance to ISAV. Given that virulent ISAV targets endothelial cells, we speculate that the kidney-specific antiviral responses of resistant fish likely aid in protecting vasculature help maintain blood filtration and volume regulation, and potentially prevent downstream cardiac stress even when blood/tissue ISAV RNA loads remain high.

### 4.5 Spleen transcriptomic profiling suggests function as an immune arsenal first, immune initiator second

While the kidney was functioning as the major early site for identifying ISAV infection and generating antiviral signaling, the spleen of resistant fish appeared to function first as a potential immune cell arsenal, while only later participating in direct antiviral signaling and ISAV recognition. The spleen functions as a peripheral lymphoid organ in fish, producing lymphocytes and antigen-specific antibodies (58). RNA-seq analysis of the spleen showed significant upregulation of mitotic and cellular generative pathways in resistant fish at 11 dpc. Increases in mitotic processes in lymphoid tissues, such as the spleen, is suggestive of the spleen functioning heavily as a hematopoietic site and generating immune cells in response to early kidney recognition of virus. In fact, the concept of infections leading to changes in hematopoiesis and immune cell generation is well established in mammals (71). Hematopoietic responses to stimuli in lymphoid tissues can happen through direct interaction with pathogens at the lymphoid tissues or interaction with pathogen-induced cytokines (IFNs, ISGs, and pro-inflammatory cytokines), with either avenue capable of inducing hematopoietic stem cell and T-cell production and differentiation (72, 73). Given all processes found to be significantly upregulated at 11 dpc in resistant fish centered around cellular division, our data support the spleen being a key organ for initial “arming up” of the host immune system through increased immune cell generation.

At 11 dpc, susceptible fish mildly upregulated spleen ISGs and IFNs compared to resistant fish. Interestingly, at 21 dpc, the trend switched, with resistant fish upregulating these ISGs more significantly. At 21 dpc, the primary differential responses as shown through RNA-seq analysis involved defense, innate immunity, stimuli response, and stress regulation. This suggests a general shift from cellular proliferation and potential immune cell progenesis to antiviral response in resistant compared to susceptible fish. This shift in gene expression is consistent with our RNA-seq data-driven hypothesis that the spleen may initially function as an immune cell proliferating hub, shifting to an antiviral response-driven role during peak infection (21 dpc) to support viral clearance.

The spleen is partially tasked with coordinating the cell-mediated antiviral response, including lymphocyte priming and production, and our results suggest that these roles may initially supersede the antiviral-response roles taken on early by the kidney and heart. To that end, the spleen of resistant fish could be better prioritizing functioning as a secondary hematopoietic organ while the spleens of susceptible fish are (1) already being significantly affected by the high viral loads, focusing on immediate anti-viral response instead of immune cell recruitment or (2) inadequately producing immune cells during early infection. Regardless, this study provides evidence that mitotic and generative processes can be associated with resistance in the spleen in early infection, with the antiviral immune response coming later (21 dpc). Of note, while one of the key drivers of variance in our transcriptomic data set overall was “Tank”, it is important to note that at the first three time points (11, 21, and 35 dpc) collected samples all came from singular tanks (see Section 2.3), thus suggesting that tank can likely be considered a proxy for time point, or “Day” in this instance (**Figure 7B**).

### 4.6 Evasion of viral antagonistic strategies may influence disease resistance

Tripartite motif (TRIM) proteins are well studied in mammalian systems as demonstrating critical activity in the early activation of the innate antiviral response (74). Nearly all described TRIM proteins contain a RING (really interesting new gene) protein domain that is capable of functioning as a mediator of ubiquitin ligase activity (75). Ubiquitin E3 ligase activity has explicitly been demonstrated in select RING-domain-containing TRIM proteins, including, but not exclusively, TRIM25 (76). Similar TRIM-associated ubiquitination activity is suspected in Atlantic salmon exposed to ISAV and infectious pancreatic necrosis virus (IPNV), suggesting mammalian–piscine parallels (77). TRIM25 interacts with the Caspase recruitment domains (CARD) of RIG-I via ubiquitination. Ubiquitination of RIG-I effectively initiates the RIG-I signaling cascade, making TRIM25 a direct mediator of the innate host antiviral response through the type I interferon system (78). The ubiquitination activity of TRIM25 and innate immune response consequences render TRIM25 a good target for viral antagonistic activity. Such is the case with the NS1 protein of Influenza Virus A (IAV, 79). The NS1 protein of IAV is a direct antagonist of TRIM25, hindering RIG-I N-terminal CARDs ubiquitin ligase activity (79). TRIM25-mediated ubiquitination is critical for the RIG-I signaling cascade, and inhibition of this by IAV NS1 is an efficacious strategy of innate immune suppression by IAV.

Interestingly, both Gervais et al. and Groves et al. identified an increase in *trim25* transcripts in resistant salmon families compared to susceptible ones (39, 40). Based on these previous findings, we explored *trim25* transcript expression levels. Herein, we partially corroborated the findings of Gervais et al. and Groves et al., finding an increase in *trim25* transcripts in the hearts (11 dpc) and kidneys (11 and 21 dpc) of fish in resistant families. In contrast, we found no difference in *trim25* transcript expression in the heart at 21 dpc. Interestingly, we found an increase in the expression of *trim25* transcripts in the spleens of susceptible fish at 11 dpc, with no differences being observed in spleens at 21 dpc. Collectively, this may suggest that *trim25* expression equalizes between resistant and susceptible fish by 21 dpc. Additionally, this suggests that spleen *trim25* expression contrasts the heart and kidney, in that it is more significantly upregulated in susceptible fish early during infection (11 dpc), which putatively indicated an earlier systemic presence of ISAV.

Similar to IAV, ISAV has been suggested to contain viral proteins that function as immune antagonists (80). Though specific activity against RIG-I ubiquitination and TRIM25 has been sparsely explored, it could be hypothesized that the ISAV genome may similarly contain a RIG-I/TRIM25 antagonist. Furthermore, our results support the hypotheses that the *trim25* gene of resistant fish (1) is being upregulated in response to viral antagonism in an effort to re-establish RIG-I pathway activation or (2) is unaffected by potential viral antagonism compared to susceptible phenotypes. Either hypothesis is also supported by the increase in IFNs and/or ISG expression observed in the heart (11 dpc) and kidney (11 and 21 dpc). Alternatively, more recent studies in mammals have found that TRIM25 may function independent of the RIG-I pathway, binding directly to IAV RNA and inhibiting viral RNA synthesis or destabilizing viral RNA prior to virion assembly (81). To that end, it could be hypothesized that comparative upregulation of *trim25* in resistant phenotypes indicates more RIG-I-independent activity, ultimately interrupting viral replication and/or virion assembly (82). While determining the precise capacity TRIM25 is acting in is outside of the scope of this study, further research into how TRIM25 is affected by and responding to ISAV infection (i.e., viral antagonistic interactions or RIG-I-independent antiviral activity) is warranted.

The importance of understanding viral antagonistic strategies cannot be understated in the context of ISAV resistance. At least two ISAV proteins, s7ORF1 and s8ORF2, have been associated with IFN and RNA interference (RNAi) pathway antagonism during early infection *in vitro* of TO cells (83, 84). Of the two proteins, s8ORF2 has been directly linked to an interaction with the Atlantic salmon genome, specifically the *SsMov10* gene. Herein, we found *SsMov10* to be significantly differentially expressed in resistant fish spleens at 21 dpc compared to susceptible via RNA-seq profiling, with no *SsMov10* transcripts detected in susceptible fish (*n* = 3) and a mean of 3,295 log-normalized transcripts found in resistant fish (**Supplementary Table S8**). There were no significant differences in *SsMov10* transcript counts in resistant and susceptible phenotypes at 49 dpc. *SsMov10* is heavily involved in host-driven RNAi. The RNAi system works sequentially, first recognizing double-stranded RNA, leading to the assembly of RNA-induced silencing complexes, and resulting in silencing of target viral mRNA. Additionally, in mammals, MOV10 also exhibits viral suppression activity against IAV by disrupting the activity of IAV nucleoprotein and subsequent assembly of the viral ribonucleoprotein complex (vRNP, 85). Assembly and activity of the vRNP is critical to viral transcription.

Similar to NS1 of IAV antagonizing the RIG-I-induced component of the Type I interferon system through TRIM25 binding, viruses have evolved mechanisms to evade the host RNAi system (86). One mechanism is through viral proteins functioning as viral suppressors of RNAi, or VSRs. Interestingly, NS1 of IAV also serves a function as a VSR (87). Similar activity to a VSR has been shown by Thukral et al. (83), with ISAV s8ORF2 suppressing the RNAi system through binding to SsMov10 and disrupting RISC assembly and maturation in early stages of *in vitro* TO cell infection (83). Thukral et al. hypothesized that negative transcriptional control of SsMov10 by s8ORF2 contributes to the collapse of host RNAi activity, and further showed that once bound to SsMov10, s8ORF2-induced RNAi suppression even increased. Of note, Thukral et al. focused *in vitro* efforts on time points prior to 11 dpc—the earliest sampling point herein (83). Given we observed an increase in *SsMov10* transcripts in resistant fish at 21 dpc, we hypothesize that *SsMov10* of resistant fish may be less permissive to s8ORF2 binding and subsequent VSR activity. To our knowledge, this is the first evidence of differential *in vivo SsMov10* activity in ISA-resistant and -susceptible phenotypes. Alternatively, and based on the findings of Zhang et al. (85), it could be hypothesized that SsMov10 is disruptive to the formation of the vRNP of ISAV as it is to IAV, and resistant fish are executing this disruption more than susceptible fish. Future efforts could verify these hypotheses by evaluating (1) downstream RNAi activity, including the SsMov10-driven, s8ORF2-prevented, RISC assembly and maturation and (2) potential disruption to vRNP assembly driven by SsMov10. Similar to NS1 of IAV, s8ORF2 is also linked to IFN antagonism, suggesting similarities between the viral proteins overall (88). Ultimately, comparative upregulation of *trim25* and *SsMov10* collectively suggests that recognition and evasion of viral immune antagonistic strategies may be a determining factor of phenotypic resistance to ISAV and/or ISA.

### 4.7 Early inflammatory response coincides with endothelial repair markers and resistance

As our findings suggested that differential suppression of viral load was not a driving determinant of phenotypic resistance, we wanted to preliminarily explore gene targets associated with endothelial damage and inflammation, as those are some of the hypothesized drivers of morbidity amid ISAV infection (12). To explore the inflammasome, we measured *tnfα*, a strong driver of the endothelial inflammatory response (89), *il1β*, a key inflammatory regulator in fish (90), and serum amyloid A (*saa*), which drives and is driven by *il1β* and *tnfα* in a positive feedback loop, at least in mammals (91). We found that resistant fish significantly upregulated proinflammatory cytokines (*tnfα* and *il1β*) and the associated inflammation-amplifier (*saa*) at 11 dpc (heart, kidney) and 21 dpc (kidney). Interestingly, excessive upregulation of *il1β* is linked to disease exacerbation and hyperinflammation in humans infected with IAV (92), while the *il1β*-mediated response in zebrafish was necessary for the initiation of regenerative processes, at least of epithelial cells (93). Our results support the latter, with an early, dynamic (but regulated) inflammatory response in the kidney and (less substantially) heart ultimately being linked to disease resistance. Interestingly, the same phenomenon was not observed in the spleen. A potential reason for this could be explained above, as the spleen seems to function primarily as an immune cell generation site at 11 dpc, then later as an antiviral responder.

Because of ISAVs’ affinity for endothelial tissues, we also explored two endothelial-associated targets: vascular endothelial growth factor (*vegf*) and hypoxia-inducible factor 1α (*hif1α*). These targets were chosen due to their potential involvement in pathways associated with endothelial tissue damage signaling, angiogenesis, and wound repair, as *hif1α* can function as a regulator of *vegf* (94–96). Both targets can also be linked back to key inflammatory markers (including *il1β* and *tnfα*) through the NF-κβ pathway (97, 98). Interestingly, *vegf* and *hif1α* were both significantly upregulated in the heart in resistant fish compared to susceptible at 11 dpc. This could suggest that while virus is moving systemically, the heart is doing a better job of recognizing damaged tissues and initiating the endothelial healing processes in resistant individuals—possibly resulting from disseminated signaling from the kidney. Interestingly, this upregulation did not maintain into 21 dpc, though it is important to note that survivorship may very well be determined by this point in infection, as peak mortality rate occurred between 11 and 21 dpc. A similar trend was observed in the kidney, with *vegf* and *hif1α* being upregulated in resistant fish, in line with the inflammatory and acute phase response genes mentioned above. This did, however, maintain into 21 dpc, suggesting that the kidney is responding consistently throughout the course of infection, at least up to 21 dpc.

## 5 Conclusions

Through this study, we have significantly increased our understanding of how salmon in the NCWMAC breeding program respond to ISAV infection after nearly two decades of selective breeding. We corroborated previous literature identifying family-based components to ISA resistance while also showing the current breeding priorities (i.e., growth and sea lice resistance) have not likely impacted ISA resistance. In doing so, we also explored the dynamics of ISA vs. ISAV resistance and determined that resistance to ISA and survivorship are not necessarily associated with resistance to ISAV infection and viral replication alone. Given that detections of virulent ISAV often require regulatory-mandated action, regardless of manifestation of clinical disease, the distinctions between differential responses associated with ISA (disease) resistance vs. those associated with ISAV (infection) resistance are important to consider. Herein we also explored systemic responses through RNA-seq of spleen tissue, which suggested that resistant fish spleens function early as an immune cell arsenal and later as a responder. Lastly, we successfully deployed dPCR technology for gene expression analysis, which highlighted early kidney responses as a differentiator for combatting ISA. We ultimately found multiple organ and phenotype-specific responses, creating a foundation for future research into what host mechanisms contribute most significantly to ISA resistance. Additional investigation targeting resistance mechanisms associated with portals of entry, such as gill, is warranted and may improve understanding for initiating the systemic responses described herein.

## Data Availability

All data generated during the challenge and subsequent analyses are available in the supplemental file. The datasets presented in this study can be found in online repositories. The names of the repository/repositories and accession number(s) can be found below: https://www.ncbi.nlm.nih.gov/, SAMN54903011– SAMN54903048.
